# The conserved WW-domain binding sites in Dystroglycan C-terminus are essential but partially redundant for Dystroglycan function

**DOI:** 10.1186/1471-213X-9-18

**Published:** 2009-02-27

**Authors:** AS Yatsenko, MM Kucherenko, M Pantoja, KA Fischer, J Madeoy, W-M Deng, M Schneider, S Baumgartner, J Akey, HR Shcherbata, H Ruohola-Baker

**Affiliations:** 1 Department of Biochemistry, Institute for Stem Cell and Regenerative Medicine, Program in Neurobiology and Behaviour, University of Washington, Seattle, WA 98195, USA; 2 Department of Genetics and Biotechnology, Ivan Franko Lviv National University, Lviv, 79005 Ukraine; 3 Max Planck Institute for biophysical chemistry, Goettingen, 37077, Germany; 4 Department of Genome Sciences, University of Washington, Seattle, WA 98195, USA; 5 Department of Biological Science, Florida State University, Tallahassee, FL 32306, USA; 6 Department of Biology, University of Copenhagen, 2100 Copenhagen, Denmark; 7 Department of Experimental Medical Sciences, Lund University, BMC B13, 22184 Lund, Sweden

## Abstract

**Background:**

Dystroglycan (Dg) is a transmembrane protein that is a part of the Dystrophin Glycoprotein Complex (DGC) which connects the extracellular matrix to the actin cytoskeleton. The C-terminal end of Dg contains a number of putative SH3, SH2 and WW domain binding sites. The most C-terminal PPXY motif has been established as a binding site for Dystrophin (Dys) WW-domain. However, our previous studies indicate that both Dystroglycan PPXY motives, WWbsI and WWbsII can bind Dystrophin protein *in vitro*.

**Results:**

We now find that both WW binding sites are important for maintaining full Dg function in the establishment of oocyte polarity in *Drosophila*. If either WW binding site is mutated, the Dg protein can still be active. However, simultaneous mutations in both WW binding sites abolish the Dg activities in both overexpression and loss-of-function oocyte polarity assays *in vivo*. Additionally, sequence comparisons of WW binding sites in 12 species of *Drosophila*, as well as in humans, reveal a high level of conservation. This preservation throughout evolution supports the idea that both WW binding sites are functionally required.

**Conclusion:**

Based on the obtained results we propose that the presence of the two WW binding sites in Dystroglycan secures the essential interaction between Dg and Dys and might further provide additional regulation for the cytoskeletal interactions of this complex.

## Background

The Dystroglycan-Dystrophin (Dg-Dys) complex has been shown to provide cells with structural integrity by forming a conduit between the extracellular matrix and the cytoskeletal network and there are lines of evidence that implicate an additional signaling role for the complex [1,2] Dystroglycan binds to extracellular matrix components, including Laminin at its N-terminus and the actin cytoskeleton via Dystrophin at its C-terminus [3,4] Defects in these interactions can result in muscular dystrophies (MD) and various epithelial cancers [5]

The characterization of the Dystrophin Glycoprotein Complex (DGC) in *Drosophila *has revealed that it possesses similar roles in muscle integrity and neuronal migration in flies as it does in humans [6] These abnormalities include age dependent muscle degeneration, reduced mobility, defects in eye development as manifested by altered photoreceptor axon path finding and photoreceptor morphology. Additionally, mutations in Dys and Dg affect cell polarity in *Drosophila *[6-8] Interestingly, some of these phenotypes are affected by the nutrition or energy metabolism in the animals [9] Recently, a reduced lifespan, as well as heart and muscle abnormalities, have been reported in *Drosophila *mutants of another component of the DGC, *δ*-sarcoglycan [10] and heart and further eye phenotypes have been observed in *Drosophila *Dys and Dg mutants [11,12]

Analogous defects observed when the Dg-Dys complex is disturbed in both flies and humans make *Drosophila *an attractive model for further studies on clarifying the cellular function of the DGC. Recent biochemical and *in vivo *structure-function analyses have revealed that a specific set of C-terminal domains are critical for the function of Dystroglycan. We have found that a putative SH3 domain binding motif but, surprisingly, not the most C-terminal Dystrophin WW domain binding motif is required for Dg function in cellular polarity in *Drosophila *[13]. However, since two potential WW binding sites exist near the Dg C-terminus it is possible that the second WW binding site can also bind Dystrophin *in vivo*, as has been shown *in vitro *[13]. In this study we dissect the roles of the two WW binding sites in the *Drosophila *Dystroglycan C-terminus *in vivo *and, interestingly, find that the sites are essential and their functions are partially overlapping.

## Results

In order to understand the regulation of Dg and its role in signaling, we have analyzed the binding motifs that are required for the function of the Dg-Dys complex in cellular polarity in *Drosophila*. The proline-rich C-terminus of Dg has several potential protein binding motifs, which suggests that it may be involved in regulating the complex and potentially may have signaling role(s). Proline-rich sequences have been shown to be the targets of several protein interaction domains involved in signal transduction. The most C-terminal PPxY motif has been established as a binding site for the WW domain of Dystrophin in humans [14-16] and in *Drosophila *by *in vitro *binding studies [6]. However, this WW domain binding site at the very C-terminus of Dystroglycan, is not essential for the function of the Dg-Dys complex in cellular polarity in *Drosophila*. An internal region of the Dystroglycan C-terminus containing a second WW domain binding site and a putative SH3 domain binding site appear to be sufficient for function in this context. We have also shown that Dystrophin can bind both the C-terminal and the internal WW domain binding sites *in vitro *[13]. We now test whether the internal WW domain binding site is essential, whether the two WW domain binding sites are redundant or whether neither is required for Dg function in *Drosophila*. To distinguish between these possibilities we used both overexpression and loss-of-function rescue analyses.

### Generation of transgenic lines expressing biochemically verified WWbs mutations

**Figure 1:**
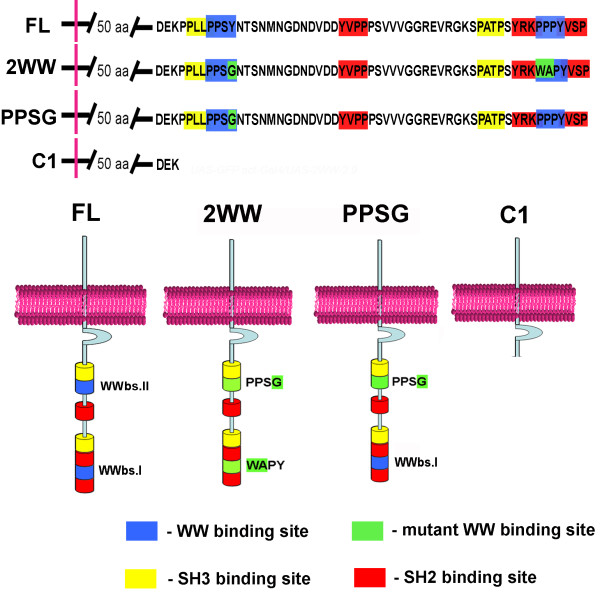
**Transgenic constructs with mutations of WW binding sites at the Dystroglycan C-terminal end. Schematic drawing of pUASp constructs with mutations in different WW binding sites**. FL – construct which encodes full length Dg, 2WW – constructs with mutations in both WW binding sites, PPSG – mutation in the N-terminal WW binding motif WWbsII PPSY → PPSG. C1 – deletion of the proline-rich C-terminus.

Previous results show that two mutations designed from computer predictions resulted in dramatic alterations in the affinity between Dg and Dys *in vitro *[13] These two mutations, predicted to abolish the WW but not the SH3 binding domain, resulted in very low binding affinities between the Dystroglycan C-terminal peptide and the Dystrophin WW domain with EF-hand region (DmWWbsI-W: Kd = 178 μM and DmWWbsII-G: Kd = 147 μM), as compared to wild type peptides (DmWWbsI: Kd = 16 μM and DmWWbsII: Kd = 46 μM). These values are comparable to the dissociation constant observed with a negative control for the assay (p53: Kd = 248 μM), suggesting that specific binding is abolished. We therefore generated transgenic lines expressing the following representative mutations: PPSG, which has a mismatch in WWbsII (PPSY → PPSG) and 2WW, which has mutations in both WW binding sites (WWbsI, PPPY → WAPY and WWbsII, PPSY → PPSG) (Figure 1). At least two independent transgenic *Drosophila *lines for each construct were obtained and analyzed. Similar results with two independent transgenic lines confirmed that the phenotype was due to the Dg mutation and not due to positional effects of the transgene inserts.

**Figure 2:**
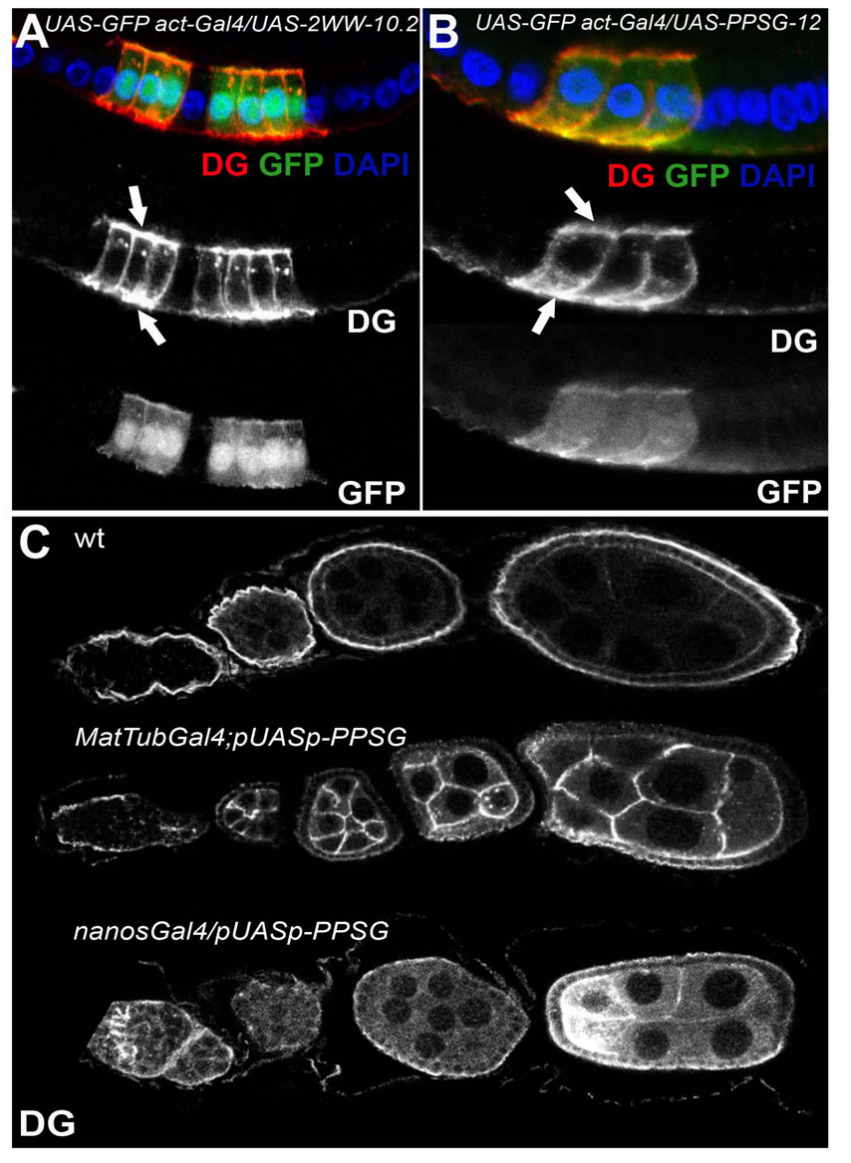
**Overexpression of Dg constructs with mutation in WW binding sites in follicle and germline cells**. **A, B**. Overexpression of 2WW (A) and PPSG (B) constructs in follicle cells marked by GFP. Dg in the wild type cells is expressed at the apical side of the follicle cell epithelium, in contrast to overexpression where Dg is localized in both apical and basal sides (indicated by arrows). C. Overexpression of the constructs in the germline cells.wt – Dg expression in wild type germline cells, MatTubGal4;pUASp-PPSG, nanosGal4/pUASp-PPSG – overexpression of transgenic constructs in germline cells. Both MatTub- and nanosGal4 have distinct expression patterns.

We first tested the ability of the transgenic constructs to produce functional forms of the Dg protein using the *Gal4/UAS *system. In order to overexpress the transgenic constructs in follicle cells we used the *hsFlp; actinFRT*<*CD2*>*FRTGal4/UAS *system in which clonal cells that overexpress the gene of interest were marked with GFP. Dg, in the wild type follicular epithelium, is located at the basal membrane (Figure 2C; WT). Overexpression of the transgenes resulted in Dg localizing to both the apical and basal sides of the follicle cells (Figure 2A, B). We also tested the expression of the constructs in germline cells using the *MatTubGal4 *and *nanosGal4 *drivers. During oogenesis, Dg is expressed at low levels in the germline (Figure 2C; WT). At stage 2–3 of oogenesis overexpression with *MatTubGal4 *shows Dg levels are substantially increased in germline cells (Figure 2C). Increased protein levels were also observed using the *nanosGAl4 *driver which showed a distinct pattern starting with high levels in the germarium, lower levels during stages 3–6 and with higher levels during later stages (Figure 2C). Similar patterns and levels of the Dg constructs were observed with all the transgenic lines analyzed in these experiments (Figure 2, Additional Figure 1, Additional Figure 4).

### WW binding site function as assayed by oocyte polarity

**Figure 3:**
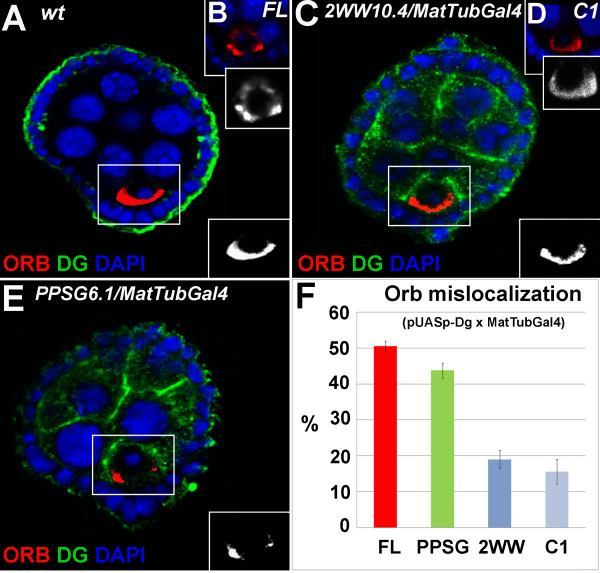
**Overexpression of pUASp with MatTubGal4 in germline disrupts the polarity marker Orb**. (Orb-red, Dg-green, DAPI – blue, separate channels for Orb are shown on the side of each corresponding picture). A. wild type (wt) stage 4 egg chamber shows normal Orb (red) localization at the posterior side of the oocyte. B. Overexpression of the pUASp-FL transgenic construct disrupts the normal Orb localization. C. Overexpression of the pUASp-2WW does not disrupt normal Orb (red) localization. Similar phenotype is seen with C1-construct that lacks the entire C-terminal region of Dg (D; Fig. 1). E. Overexpression of pUASp-PPSG constructs disrupts oocyte polarity indicated by mislocalization of Orb which has an abnormal side location, F. Percentage of Orb mislocalization as the result of overexpression of different pUASp-Dg constructs. (FL, 49 ± 2, PPSG 44 ± 2, 2WW 19 ± 2, C1 16 ± 3).

To analyze whether the Dg mutant forms are functional in oocyte polarity, we expressed mutant and wild type Dg constructs in germline cells using a germline specific driver (*MatTubGal4*), and examined oocyte polarity using Orb protein as a marker. Orb is a member of the cytoplasmic polyadenylation element binding (CPEB) family of RNA-binding proteins that are implicated in local protein synthesis [17]. In *Drosophila *oogenesis Orb co-localizes with the microtubule organizing center (MTOC), which is localized to the anterior of the oocyte during stage 1, and then moves to the posterior by stage 3. Between stages 3 and 6, Orb is clearly localized to the posterior of the oocyte, making it an excellent marker to analyze the polarity of the oocyte (Figure 3A, 4A). Absent or mislocalized Orb during these stages indicates a failure to establish early oocyte polarity.

**Figure 4:**
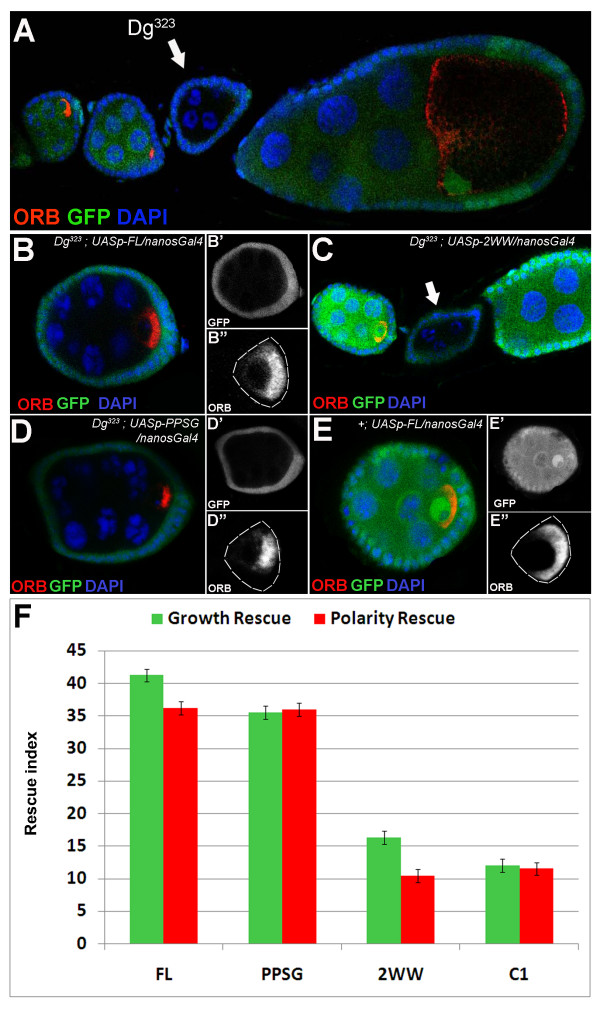
**Rescue of Dg loss-of-function germline clones with expression of pUASp-Dg constructs**. Orb (Red); GFP (Green), DAPI (Blue) A', B', D' GFP of the corresponding stages shown in a separate channel; A", B", D" Orb staining of the corresponding stages shown in a separate channel with dotted lines which indicates the border of the oocyte. A. Dg loss-of-function germline clones (black, white arrow; *hsFLP; FRT42D Dg*^323^) are arrested prior to stage 6 and have disrupted oocyte polarity (absent or mislocalized Orb). B. Expression of *pUASp-FL *with the nanos-Gal4 driver in Dg clones partially rescues oocyte polarity in arrested clones stages 3–6 as indicated by proper localization of Orb to the posterior of 36% of the oocytes; (*hsFLP; FRT42D Dg*^323^*; P(w+:nanosGal4:VP-16)Ab-2/pUASp-FL) C*. Expression of pUASp-2WW with the *nanos-Gal4 *driver in *Dg *clones does not rescues oocyte polarity in arrested clones stages 3–6 (arrow) [as indicated by development arrest and absent Orb marker; (*hsFLP; FRT42D Dg*^323^*; P(w+:nanosGal4:VP-16)Ab-2/pUASp-2WW*)] *D*. Expression of *pUASp-PPSG *with the *nanos-Gal4 *driver in *Dg *clones rescues oocyte polarity in arrested clones stages 3–6 [as indicated by proper localization of Orb to the posterior of the oocyte; (*hsFLP; FRT42D Dg*^323^*; P(w+:nanosGal4:VP-16)Ab-2/pUASp-PPSG*)]*E*. Wild type egg chamber with posterior Orb localization (+/+; *P(w+:nanosGal4:VP-16)Ab-2/pUASp-FL) F*. FL, PPSG are able to rescue Dg loss-of-function phenotypes, while 2WW and C1 do not (Red: rescued polarity index, Green: rescued growth index).

We have previously shown that overexpression of the wild type form of *Drosophila *Dystroglycan (FL = full length) is sufficient to generate oocyte polarity defects [13] (Figure 3B). When FL is overexpressed in the germline, Orb becomes mislocalized, surrounds the entire oocyte nucleus, or accumulates in a clump to one side of the oocyte instead of localizing to the posterior. Therefore, Dystroglycan, when expressed at elevated levels in germline cells, is sufficient to disrupt oocyte polarity. Overexpression of the full length form of Dg with the *tubGal4 *driver causes semi-lethality (data not shown). Similarly, in vertebrates overexpression of Dg has been shown to cause defects in neuromuscular junctions [18,19]. We used the overexpression oocyte polarity assays to test whether either of the WW domain binding sites is essential for Dystroglycan function.

To test the function of WWbsI *in vivo *we overexpressed the PPSG mutant protein in germline cells using the *MatTubGal4 *driver and determined the localization of the early oocyte polarity marker Orb. As discussed, in wild type cells Orb marks the localization of the microtubule organizing center and is localized to the posterior side of the oocyte during stages 3–6 (Figure 3A). Overproduction of the PPSG protein results in the mislocalization of the usually posterior Orb marker. In mutants Orb surrounds the oocyte nucleus or localizes to the sides of the oocyte nucleus in 44 ± 2% of 3–6 stage oocytes (n = 147, Figure 3E–F). The level of this defect is similar to the one observed with the FL construct [6,13], which contains both WW binding sites (Figure 1; Figure 3B, D; 49 ± 2%, n = 80). These data suggest that disturbing the second WW binding site at the Dg C-terminus does not dramatically affect the functionality of the protein; similar to FL construct, when overexpressed it still is sufficient to disturb the oocyte polarity.

In contrast to the FL and PPSG constructs, overexpression of a 2WW mutant construct did not result in a high percentage of Orb mislocalization (Figure 3C, F, 19 ± 3%, n = 123). With 2WW overexpression, Orb, in most cases, was localized to the posterior of the oocyte (Figure 3C). The frequency of mislocalization with the 2WW construct, in which both WW binding sites were mutated was similar to that of the C1 construct which lacked all the C-terminal binding sites (Figure 1, Figure 3D, F, 16 ± 2%, n = 86).

These data, in combination with our previous data [13] show that a single mutation in WWbsII or the lack of WWbsI does not result in dramatic defects in Dg activity in this sufficiency assay measuring the oocyte polarity. However, simultaneous mutations in both WW binding sites dramatically reduce the function of Dystroglycan in this assay.

### One WW binding site is required for Dystroglycan function

We also tested the function of the WW binding site mutants in rescue experiments by expressing the transgenes in a Dg loss-of-function background. *Dg*^323 ^germline mutant clones are arrested prior to stage 3–4 and have mislocalized or missing Orb protein (Figure 4A). We have previously shown that these defects are partially rescued by wild type (full-length) Dg expression [13] (Figure 4, 36–40% rescue). Full rescue is not expected since the Dg^323 ^deletion also affects a newly described neighboring gene mRpl34 (Additional Figure 3) and recent data implies that the level of nutrients and energy metabolism in the animal may affect cellular polarity [9]. To test if our mutant constructs were capable of rescuing the developmental arrest and the defects in oocyte polarity on the same level as the Dg full-length construct, we expressed them using the germline driver *nanosGal4 *and calculated the percentage of loss-of-function clones with rescued growth and polarity. Using this assay we tested whether the Dg WWbs mutations were capable of a similar level of rescue as full-length Dg. If the Dg mutant with both WW binding sites mutated (2WW, Figure 1) could rescue the *Dg*^323 ^phenotype in oocyte polarity at the same level as wild type Dg, we conclude that neither of the WW binding sites in *Drosophila *is required for Dg activity. On the other hand, if Dg with two WWbs mutations cannot rescue, we conclude that both or just the internal WW binding site is essential for Dg activity (we have already shown that the C-terminal WWbs is not essential [13]). As discussed above, to distinguish between these possibilities, we have generated a single mutation in WWbsII (PPSG, Figure 1) and will test whether this mutant still has the full length Dg activity in the loss-of-function rescue assays.

Similar to the full length Dg (FL, Figure 4B), the PPSG mutant constructs were capable of partially rescuing the Dg mutant phenotype (Figure 4D). Loss-of-function clones with expression of FL (Figure 4B-B") and PPSG (Figure 4D-D") had similar levels of posterior localization of the polarity marker, Orb (Figure 4F, FL: 36 ± 0.5% n = 52; PPSG: 41% n = 22). These mutants were also capable of restoring the developmental arrest phenotype by showing a higher percentage of loss-of-function clones that were older than stage 4–6 (Figure 4F, FL: 47 ± 8% n = 55; PPSG 38% n = 21). In contrast, 2WW was unable to rescue (2WW rescued at the level of the C1 mutant that lacks most of the Dg C-terminus; [13]; Figure 1.). Dg loss-of-function clones with expression of 2WW and C1 showed lower percentages of normal polarity (Figure 4F; 2WW: 12 ± 0.6% n = 66; C1: 9% n = 22) and growth rescue (Figure 4F; 2WW: 19 ± 2% n = 66; C1: 13%) than FL or PPSG constructs (Figure 4F). This result indicates that at least one WW binding site is required for normal function of Dg but a mutation in only one of the sites does not alter the functionality of Dg protein dramatically.

Since a single WWbsII mutation or a WWbsI deletion does not cause a severe loss of Dg activity but the double mutant does, we conclude that the two binding sites act, at least partially in a redundant manner in oocyte polarity and growth assays.

### WW binding sites are highly conserved

**Figure 5:**
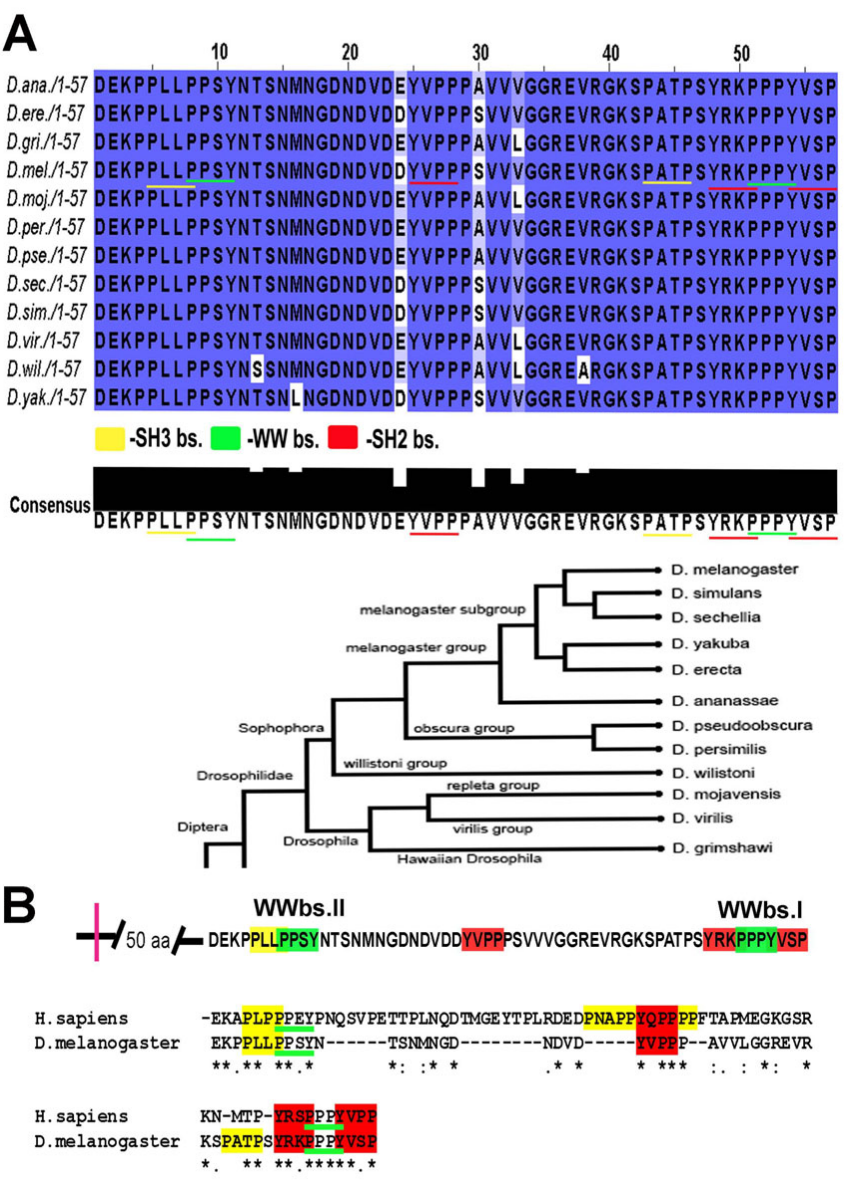
**Both WW binding sites are conserved in all 12 species of *Drosophila***. A. Amino acid sequence alignment of the C-terminal end from 12 species of *Drosophila *using the computer program ClustalW shows absolutely no variation between both WW binding sites. B. Both WW binding sites are highly conserved between humans and *Drosophila*.

Since both WW binding sites proved to be important in our *in vivo *experiments we wanted to know if the importance of these sites has been preserved among the interspecies population. To analyze the conservation of WW binding sites, we tested for variability in the sequence of those sites among all *Drosophila *species. For this purpose, using the ClustalW program, we aligned the *Dystroglycan *sequences of the 12 species of *Drosophila *obtained from the GBrowse database. The alignment analysis indicates that the two WW binding sites are fully conserved among all 12 *Drosophila *species (Figure 5A). Some variation in the nucleotide sequences of the WW binding sites were observed between the species, however these changes did not lead to amino acid sequence changes (Additional Figure 2). Furthermore, both Dg WW binding sites were also conserved between *Drosophila *and humans (Figure 5B).

In order to better understand patterns of polymorphisms in human Dystroglycan (*DAG*), and, in particular, the WW domains, we sequenced a 348 bp fragment spanning the region of interest in 88 samples from six geographically diverse human populations. In total, only one segregating site was identified among the 176 chromosomes sequenced (table 1) and none were identified in either of the WW domains. The estimated nucleotide diversity (defined as the average number of pairwise differences between two randomly selected chromosomes per nucleotide) in the combined sample is 3.24 × 10^-5^. In contrast, the average nucleotide diversity of 322 genetic regions that were sequenced in a panel of 23 European-Americans and 22 African-Americans is 8.53 × 10^-4^, suggesting that the sequenced region of *DAG *is under significant functional constraint. These data suggest that during evolution both WW binding sites have been important and therefore are preserved among species.

**Table 1:** Summary statistics of sequencing data.

**Population**	**N^a^**	**S^b^**	**θ_W_^c^**	**π^d^**
CEPH	40	0	0	0
Han Chinese	40	0	0	0
Middle East	20	0	0	0
Pygmy	20	0	0	0
South Africa	16	0	0	0
South America	20	1	5.26 × 10^-3^	2.73 × 10^-4^
South East Asia	20	0	0	0

Total	176	1	6.49 × 10^-5^	3.24 × 10^-5^

^a ^Number of chromosomes

^b ^Number of segregating sites

^c ^Watterston's theta per bp

^d ^Nucleotide diversity per bp

## Discussion

The functional redundancy of the WW binding sites poses interesting questions: have both binding sites survived through evolution to protect organisms from the mutations in an essential complex or does each binding site have a specific function in different tissues and/or developmental stages. Mutations in the DGC cause muscular dystrophies; however only mutations in Dystrophin, but not Dystroglycan per se, are associated with known types of muscular dystrophies in vertebrates. In mice, mutations in Dystroglycan are embryonic lethal, which suggests that Dg is an essential gene and, perhaps the redundant Dystrophin binding sites in Dystroglycan provide an additional means for DGC regulation.

The comparative sequence analysis of *Drosophila *and human WW binding motifs revealed very high conservation. However, each WWbs resides in a specific protein micro-environment, which may suggest that each site has specific binding partners. The previously performed genetic screens for modifiers [20] of Dg and Dys showed that the Dg-Dys complex interacts with components of different signaling pathways and components involved in cell/neuronal migration, cytoskeletal rearrangement and muscle development. This suggests that the Dg-Dys complex might be a major hub that regulates transfer of extracellular information to the cytoskeleton. Therefore it will be important in the future to test if WW binding sites have specific and independent biological functions in different tissues. This kind of analysis is likely to provide insights into the specific functions of the Dg-Dys complex and serve as a basis for the development of novel therapeutic approaches for the treatment of muscular dystrophy.

## Conclusion

We have investigated the role of the WW binding sites at the C-terminus of Dystroglycan protein and found that both sites may bind to the WW+EF hand domain of Dystrophin. Our previous studies [6,13], indicate that WWbsI and WWbsII both can bind Dystrophin protein *in vitro*. To test whether both WW binding sites can function and are required *in vivo *we generated two transgenic mutants: 2WW, which has mutations in both WW binding sites (WWbsI, PPPY → WAPY and WWbsII, PPSY → PPSG), and PPSG, which has a mismatch in WWbsII (PPSY → PPSG). We used the establishment of early oocyte polarity as an assay to verify the functionality of WWbsI and WWbsII. Importantly, the data show that while each WW binding site mutation yields to close to normal Dg function, the double WWbs mutation has lost Dg C-terminal activity. These data suggest that at least one WWbs is required for full Dg function *in vivo *and that the two sites may be partially redundant.

## Methods

### Fly Stocks

*Drosophila melanogaster *stocks were raised on standard cornmeal/yeast/agar medium at 25°C. For overproduction of pUASp-Dg in the germline, we used the following: *NGT40*; *P*(*w*^+^:*nanosGal4*:*VP-16*)*Ab-2 *[21,22] and *Mat*-α*4-TubGal4-VP16/CyO *[23]. For overproduction of pUASp-Dg in the follicle cells, we used *hsFlp; act < FRT-CD2-FRT < Gal4; UAS-GFP*[24]. For generation of *Dystroglycan *clones, we used *FRT42D-Dg*^323^*/CyO *(*Dg*^323 ^is a *Dystroglycan *loss-of-function mutant with a 3324 bp deletion between bp 32,345 and 35,669 of DS03910 [7] disrupting the Dg 5' region and the adjacent mRPL34 gene; Additional Figure 3) and *hsFLP;FRT42D Ubi-GFP/CyO*. For overproduction of pUASp-Dg in a *Dystroglycan *mutant background, we used *FRT42D-Dg*323/*CyO; P*(*w nos-Gal4*:*VP16*)*A4-2 III*, and *hsFLP; FRT42D Ubi-GFP/CyO; pUASp-Dg/TM3 *(pUASp-Dg refers to all *Dystroglycan *constructs: FL, C1, 2WW, PPSG). Two deletions in the *Dystroglycan *region exist; *Dg*^248 ^(11985709:11986494) whose breakpoints are 333 bp downstream of the *Dg *transcription start site (11986042) and 3 bp upstream of the mRpL34 start codon (11986498) and *Dg*^323 ^(11983340:11986664) whose breakpoints are 2.7 kb downstream of the *Dg *transcription start site and 166 bp downstream of the mRpL34 start codon (Additional Figure 3). We also used: dg043 [25].

### Generation of pUASp-Dg Transgenic Animal

Full length and modified *Dystroglycan *PCR products that can be expressed in the germline were synthesized from the template LD11619. pUASp-FL and pUASp-C1 constructs used in this work have been described previously [13]. To generate a construct with mutated WWbsII (pUASp-PPSG) LD11619 was used as a template with the following primers: 5'-GGGGTACCAACATGAGATTCCAGTGGTTCT-3' 5'-GCTCTAGATTATGGCGACACACATA-TGGCGGT-3'. The PCR products were digested with KpnI and XbaI and cloned into the pUASp vector [26]. The constructs were injected into embryos to obtain at least two independent stable transformant lines. Injections were done by Rainbow Transgenic Flies, Inc. (California, USA).

### Overproduction of Dystroglycan in the Germline and Follicle Cell

For overproduction in germline cells, balanced *pUASp-Dg/Mat*-α*4-TubGal4-UP16/CyO or P*(*w*^-^: *nanosGal4*:*VP-16*)*Ab-2 *animals were raised in yeasted vials at 25°C for 3 days before dissection and analysis. For overproduction in the follicle cells, *hsFlp; UAS-GFP act *<*FRTCD2FRT *<*Gal4/pUASp-Dg *animals were heat-shocked at 37°C for 1 h, raised in yeasted vials at 25°C for 3 days before dissection and analysis. All pUASp-Dg constructs used were crossed to these three Gal4 drivers to test for proper overproduction of protein and correct localization of protein to the membrane in the germline and somatic cells. The following pUASp-Dg lines were used for germline analysis: FL-1, 5; C1-1, -2; 2WW-10.2, -5.6, -13, 15.4; PPSG-11.1, -12.5, -6.3, -13.4. For the rescue experiments the following lines were used: FL-1, -2, -5; C1-1, -2; 2WW-10.4, -13, -15.6; PPSG-11.4, 11.1.

### Antibody Staining Procedures

*Drosophila *ovaries were dissected rapidly in PBS and fixed in 4% paraformaldehyde for 10 minutes. The antibody staining procedure was the same as described previously [13]. The following primary antibodies were used at the following designated dilutions: rabbit anti-Dystroglycan (1:3000 [7]), mouse anti-Orb (1:20; Developmental Studies Hybridoma Bank), the following secondary antibodies were used at the designated dilutions: Alexa 488 anti-rabbit and Alexa 568 anti-mouse (1:500; Molecular Probes).

### Western Blot and densitometry analyses

Sample preparation and SDS-PAGE have been described previously [13]. Bio-Rad ready-made 4–20% polyacrylamide gels were used for protein separation. Proteins were transferred to polyvinylidene difluoride (PDVF) membranes (Immobilon) using a semi-dry transfer apparatus (Bio-Rad). Primary affinity purified anti-Dg antibodies were used at 1:30,000 dilutions. Goat anti-rabbit HRP conjugated antibodies (Bio-Rad) were used as detection reagents at 1:10,000 dilutions. Proteins were visualized via enhanced chemiluminescence (Millipore). Densitometry analysis was performed with the public domain NIH IMAGEJ program (developed at the US National Institutes of Health and available on the Web at ). Scans of immunoblots determined to be in the linear range (i.e. twice the amount of protein correlated with twice the signal seen on photographic film) were used as sources for analysis.

### Sequence alignment

Sequences of 12 species of *Drosophila *were obtained from the FlyBase genome database. Sequence alignment was done using software ClustalW designed by the European Bioinformatics Institute .

### DNA samples used for sequencing

We sequenced a 348 bp fragment of *DAG *that includes both WW domains in DNA samples from 88 humans representing six populations. Samples were obtained from the Coriell Institute for Medical Research Cell Repositories (Camden, NJ, USA). Coriell repository numbers for these samples are as follows: CEPH European-American (NA06990, NA07019, NA10830, NA10831, NA07348, NA07349, NA10842, NA10843, NA10844, NA10845, NA10848, NA10850, NA10851, NA10852, NA10853, NA10854, NA10857, NA10858, NA10860, NA10861, NA17201) Han Chinese of L.A. (NA17733 – NA17747, NA17749, NA17752 – NA17757, NA17759, and NA17761), Middle East (NA17041 – NA17050), Pygmy (NA10469 – NA10473, NA10492 – NA10496), South Africa (NA17319, NA17341 – NA17348), South America (NA17301 – NA17310) and South East Asia (NA17081 – NA17090). We compared patterns of polymorphism to 322 genes that were sequenced as part of the SeattleSNPs project [27].

### DNA sequencing and statistical analysis

Sequencing primers were designed with primer3 (; primer sequences available upon request). We used standard PCR-based sequencing reactions using Applied Biosystem's Big Dye sequencing protocol on an ABI 3130xl. Sequence data was assembled using Phred/Phrap [28,29] and the alignments were inspected for accuracy with Consed [30,31]. Polymorphisms were identified with PolyPhred 4.0 [32]. All polymorphic sites were manually verified and confirmed by sequencing the opposite strand. Standard measures of nucleotide diversity, including θ_W _and π were calculated as previously described [27].

## Authors' contributions

ASY conception, design, acquisition, analysis and interpretation of the data, drafting the manuscript. MMK conception, design, acquisition, analysis and interpretation of the data. HRS drafting the manuscript. MP conception, design, acquisition, analysis and interpretation of the data, revising the manuscript. KAF acquisition, analysis and interpretation of the data. JM acquisition, analysis and interpretation of the data. WMD conception and interpretation of the data. MS conception and interpretation of the data. SB conception and interpretation of the data. JA conception, design and interpretation of the data, drafting the manuscript. HRB conception, design and interpretation of the data, drafting the manuscript. All the authors have read the article and accepted the final manuscript.

## Supplementary Material

Additional file 1 **Figure 1**. Overexpression of Dg constructs with mutation in WW binding sites in follicle and germline cells. A, B. Overexpression of 2WW (A) and PPSG (B) constructs in follicle cells marked by GFP. Dg in the wild type cells is expressed at the apical side of the follicle cell epithelium, in contrast to overexpression where Dg is localized in both apical and basal sides (indicated by arrows). To compare the expression levels of different constructs and insertions the intensities of Dg expression was compared to the intensity of the GFP signal in the same cell. The observed mean intensity ratios are similar in the two constructs (2WW = 1.2, PPSG = 1.1), suggesting that the differences observed between these two conatructs in oocyte polarity assay are not due to dramatically different levels of expression. C. Overexpression of the constructs in the germline cells.wt – Dg expression in wild type germline cells, MatTubGal4; pUASp-WW, nanosGal4/pUASp-WW – overexpression of transgenic constructs in germline cells. Both MatTub- and nanosGal4 have distinct expression patterns. Click here for file

Additional file 4 **Figure 4**. Western blot analysis of Dg protein in wild type, DgO43, 2WW and PPSG ovaries and whole animals show the following Dg intensities compared to OregonR (WT): DgO43 [25] = 0.4, 2WW = 1.3, PPSG = 1.2. The specific bands that correspond to different Dg forms can be seen at ~180 (two bands), 110 and faintly at 70 kD. A presumable degradation product can be seen below 25 kD. Increased band intensities can be seen with the 110 kD band and most notably with the higher 180 kD species. Band intensities were normalized to actin and samples were run on a gradient 4–20% gel. Click here for file

Additional file 3 **Figure 3**. The genomic region of the *Dystroglycan *gene. The genomic regions that are deleted in the *Dystroglycan *mutant alleles *Dg*^323 ^and *Dg*^248 ^are indicated as black bars. Click here for file

Additional file 2 **Figure 2**. Comparative analysis of Dg C-terminus nucleic acid sequences in 12 species of *Drosophila*. Click here for file
