# Supplementary figures and images for: The conserved WW-domain binding sites in Dystroglycan C-terminus are essential but partially redundant for Dystroglycan function
Source: BMC Dev Biol. 2009 Feb 27;9:18. doi: 10.1186/1471-213X-9-18 (PMC2660313; doi:10.1186/1471-213X-9-18)

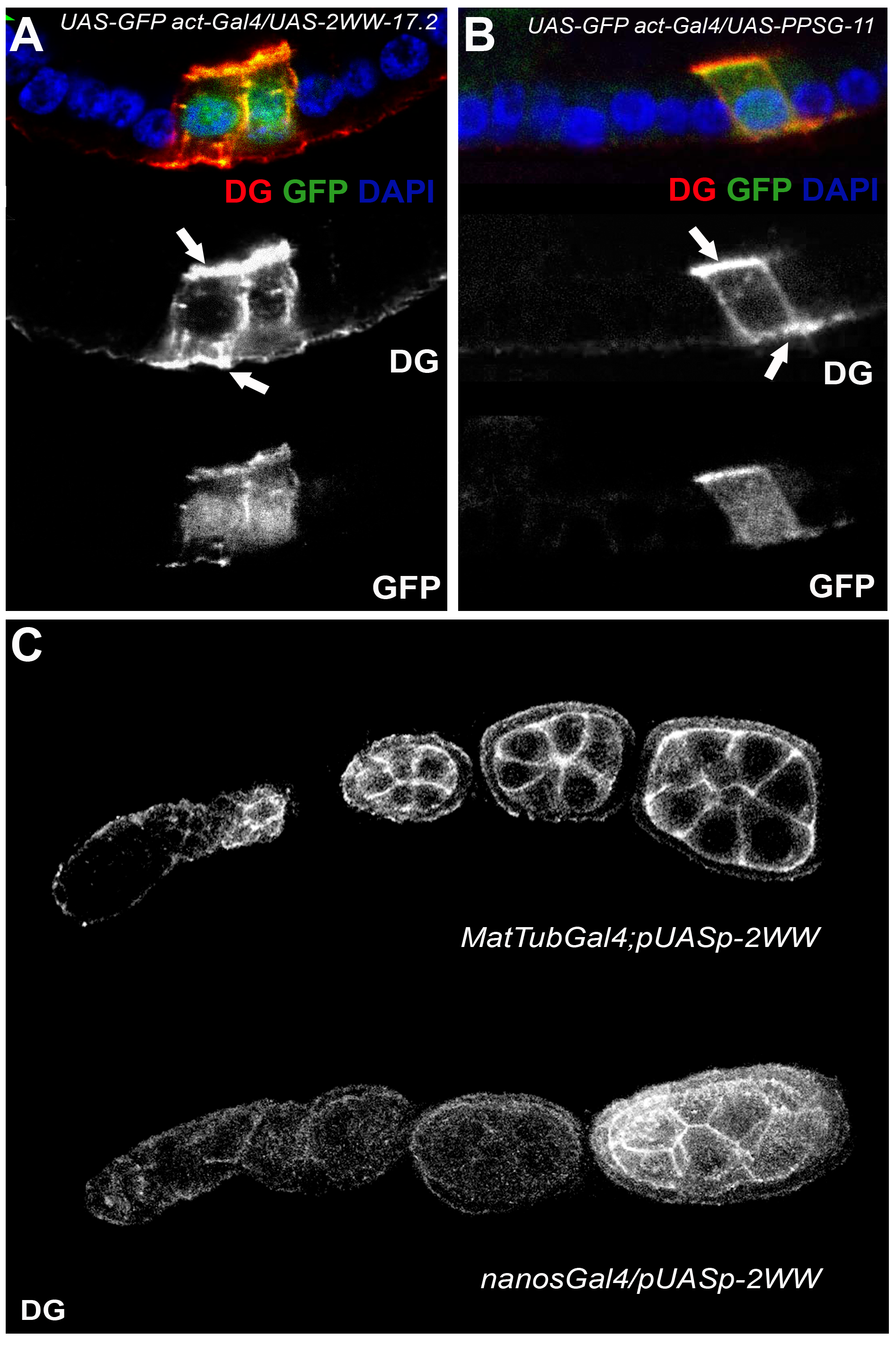

Supplement: Additional file 1 — Figure 1. Overexpression of Dg constructs with mutation in WW binding sites in follicle and germline cells. A, B. Overexpression of 2WW (A) and PPSG (B) constructs in follicle cells marked by GFP. Dg in the wild type cells is expressed at the apical side of the follicle cell epithelium, in contrast to overexpression where Dg is localized in both apical and basal sides (indicated by arrows). To compare the expression levels of different constructs and insertions the intensities of Dg expression was compared to the intensity of the GFP signal in the same cell. The observed mean intensity ratios are similar in the two constructs (2WW = 1.2, PPSG = 1.1), suggesting that the differences observed between these two conatructs in oocyte polarity assay are not due to dramatically different levels of expression. C. Overexpression of the constructs in the germline cells.wt – Dg expression in wild type germline cells, MatTubGal4; pUASp-WW, nanosGal4/pUASp-WW – overexpression of transgenic constructs in germline cells. Both MatTub- and nanosGal4 have distinct expression patterns. [file 1471-213X-9-18-S1.tiff]

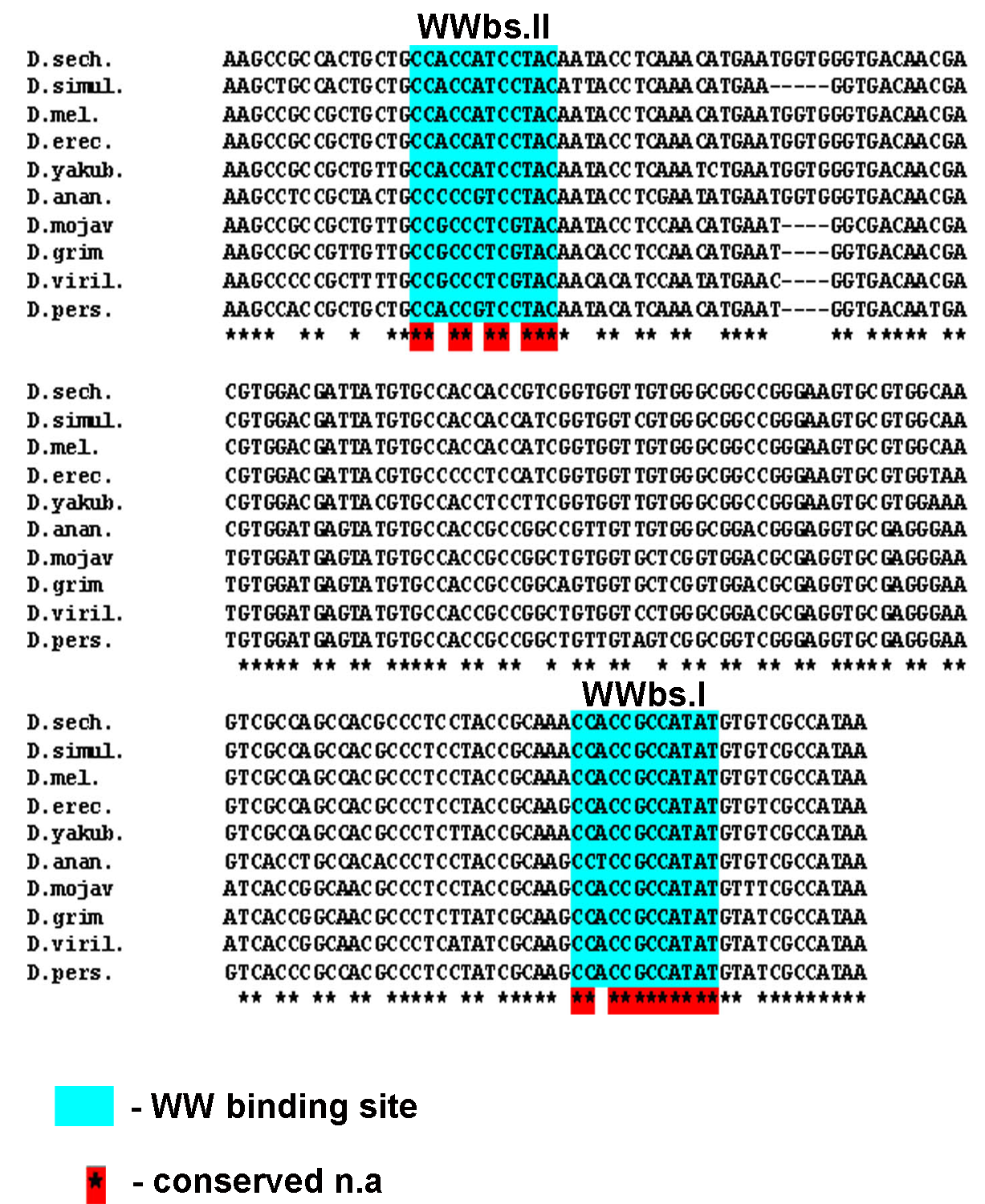

Supplement: Additional file 4 — Figure 4. Western blot analysis of Dg protein in wild type, DgO43, 2WW and PPSG ovaries and whole animals show the following Dg intensities compared to OregonR (WT): DgO43 [25] = 0.4, 2WW = 1.3, PPSG = 1.2. The specific bands that correspond to different Dg forms can be seen at ~180 (two bands), 110 and faintly at 70 kD. A presumable degradation product can be seen below 25 kD. Increased band intensities can be seen with the 110 kD band and most notably with the higher 180 kD species. Band intensities were normalized to actin and samples were run on a gradient 4–20% gel. [file 1471-213X-9-18-S4.tiff]

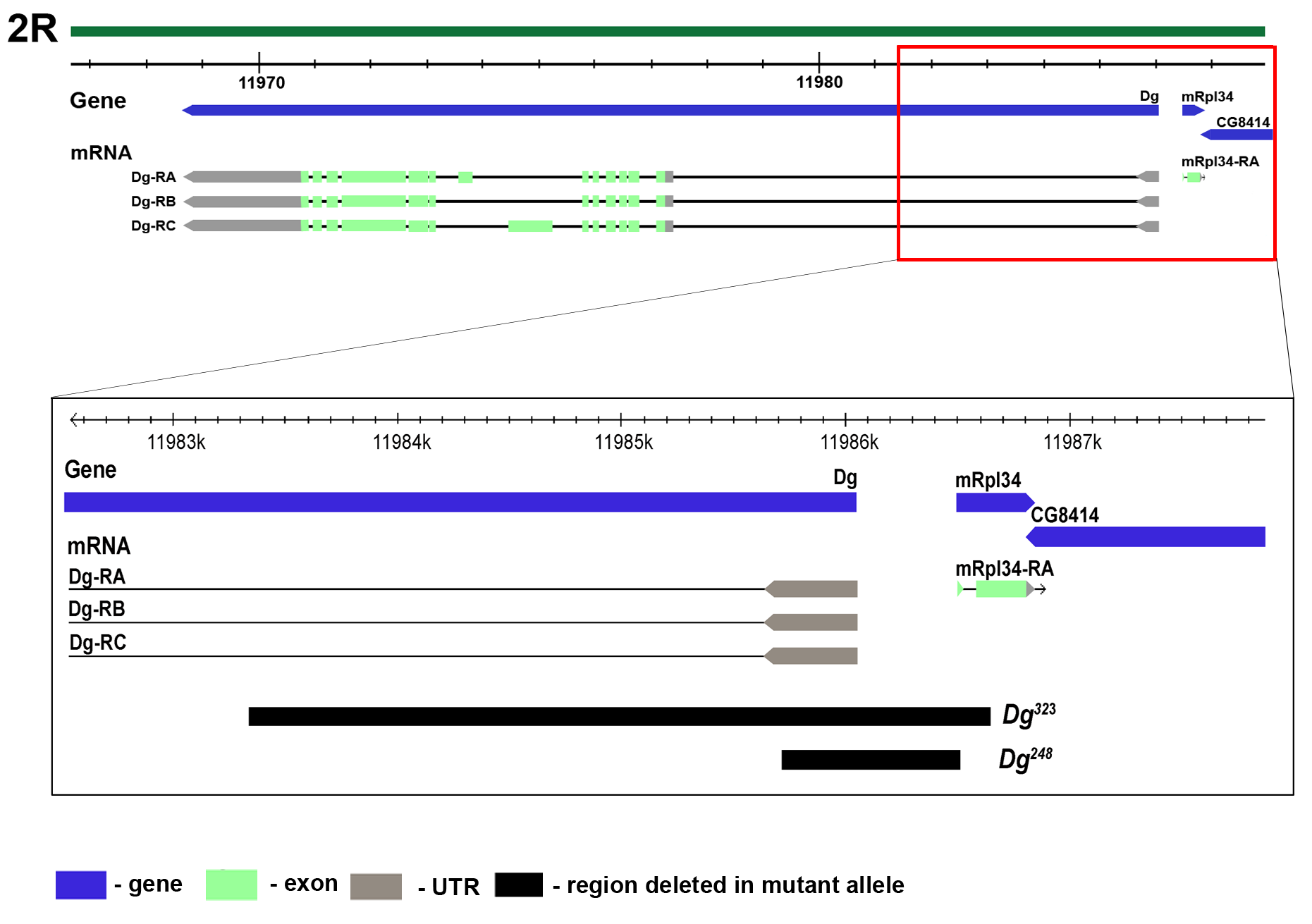

Supplement: Additional file 3 — Figure 3. The genomic region of the Dystroglycan gene. The genomic regions that are deleted in the Dystroglycan mutant alleles Dg323 and Dg248 are indicated as black bars. [file 1471-213X-9-18-S3.tiff]

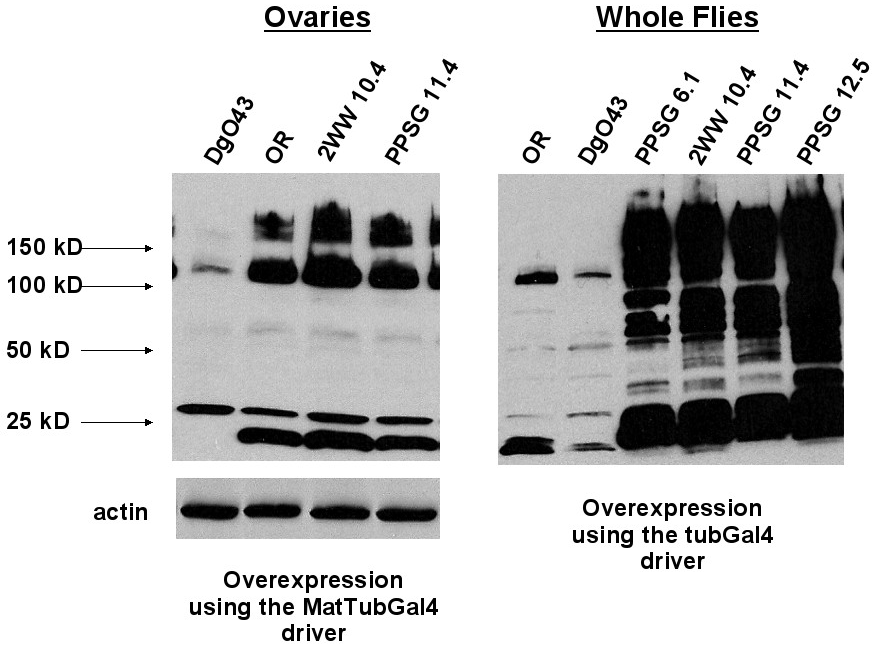

Supplement: Additional file 2 — Figure 2. Comparative analysis of Dg C-terminus nucleic acid sequences in 12 species of Drosophila. [file 1471-213X-9-18-S2.tiff]
